# Molecular and Cellular Effects of Microplastics and Nanoplastics: Focus on Inflammation and Senescence

**DOI:** 10.3390/cells13211788

**Published:** 2024-10-29

**Authors:** Faiza Mahmud, Drishty B. Sarker, Jonathan A. Jocelyn, Qing-Xiang Amy Sang

**Affiliations:** 1 Department of Chemistry and Biochemistry, Florida State University, Tallahassee, FL 32306-4390, USA; fm23@fsu.edu (F.M.); ds22@fsu.edu (D.B.S.); jaj21g@fsu.edu (J.A.J.); 2 Institute of Molecular Biophysics, Florida State University, Tallahassee, FL 32306-4380, USA

**Keywords:** microplastics, nanoplastics, reactive oxygen species, cytotoxicity, inflammation, senescence, cellular aging, DNA damage, environmental pollutants, human health

## Abstract

Microplastics and nanoplastics (MNPs) are ubiquitous environmental contaminants. Their prevalence, persistence, and increasing industrial production have led to questions about their long-term impact on human and animal health. This narrative review describes the effects of MNPs on oxidative stress, inflammation, and aging. Exposure to MNPs leads to increased production of reactive oxygen species (ROS) across multiple experimental models, including cell lines, organoids, and animal systems. ROS can cause damage to cellular macromolecules such as DNA, proteins, and lipids. Direct interaction between MNPs and immune cells or an indirect result of oxidative stress-mediated cellular damage may lead to increased production of pro-inflammatory cytokines throughout different MNP-exposure conditions. This inflammatory response is a common feature in the pathogenesis of neurodegenerative, cardiovascular, and other age-related diseases. MNPs also act as cell senescence inducers by promoting mitochondrial dysfunction, impairing autophagy, and activating DNA damage responses, exacerbating cellular aging altogether. Increased senescence of reproductive cells and transfer of MNPs/induced damages from parents to offspring in animals further corroborates the transgenerational health risks of the tiny particles. This review aims to provoke a deeper investigation into the notorious effects these pervasive particles may have on human well-being and longevity.

## 1. Introduction

The widespread utilization and crude disposal of plastic products have contributed to an alarming escalation of the existence of microplastics and nanoplastics (MNPs) in the environment [1]. The intrinsic properties of organic polymers provide an efficient synthesis and inexpensive production, resulting in the expanding global production of plastic products and their subsequent diffusion across the globe. However, this extensive use is accompanied by a proportional flow of nonbiodegradable residue and waste [2]. Although measures have been taken all over the world to encourage the recycling of polymers through methods of assimilation and repurposing to reduce the generation of plastic waste, insufficient plastic disposal persists worldwide [3,4]. These have accumulated into the generation of a whopping 464 million tons of plastic waste across the world every year [5,6]. This intensifying presence of waste in the environment signifies a concerning shift in the threat of long-term plastic prominence in our environment, solidifying prolonged human exposure to plastic as no longer just a concern, but a reality that warrants immediate attention.

Despite extensive sanitation measures in society, humans remain at risk of increased exposure to micro- and nanoplastic (MNP) particles, not only through direct ingestion but also via inhalation of elevated concentrations present in the air. Regardless of the pathway, exposure remains an inevitability within modern society, with even ostensibly healthy subjects across studies being documented as having polymeric particles ranging from 4–100 µm present within their placenta [7,8], stool [9], lungs [10], and liver [11]. Chronic exposure of human cells to microparticles can lead to elevated production of reactive oxygen species (ROS) and aggravated oxidative stress [12,13]. Extensive oxidative stress within human cells has been linked to subsequent inflammation [14], pulmonary disease [15,16], and carcinogenesis [17]. Oxidative stress is also intricately linked to cellular senescence, which accounts for significant cellular damage and arrest [18,19], increased vulnerability to carcinogenic promotion [20], immune degradation [21], and cognitive decline [22,23,24]. There is evidence of upregulated inflammatory markers in cells affected by microplastics and nanoplastics as well [25,26]. Persistent inflammation can lead to cell cycle arrest, thereby inducing cellular senescence [27]. This potential danger to human cells at the cellular and molecular levels necessitates further research to develop knowledge and effective solutions.

Our review aims to illustrate the extent of existing information regarding the impacts of micro and nanoplastic exposure on human cell inflammation and senescence, ideally providing a precedent for further research and insight regarding the subject of growing importance.

## 2. Molecular Impacts of Micro- and Nanoplastic Exposure in Human Cells

### 2.1. Generation of Reactive Oxygen Species (ROS)

Free radicals refer to atoms or groups of atoms that contain one or more unpaired electrons and show reactivity, including potential for oxidative reactions [28]. In living systems, reactive oxygen species (ROS) are the radicals that originate from single oxygen or free radicals such as superoxide radicals or non-radical molecules such as hydrogen peroxide. Micro- and nanoplastics can work as electron donors capable of producing superoxide radicals [25,29,30]. By reaction with metals like iron, copper, etc., hydrogen peroxide can convert into hydroxyl radicals (Fenton reaction) or superoxide radicals as well (Haber–Weiss reaction) [31].

**Figure 1:**
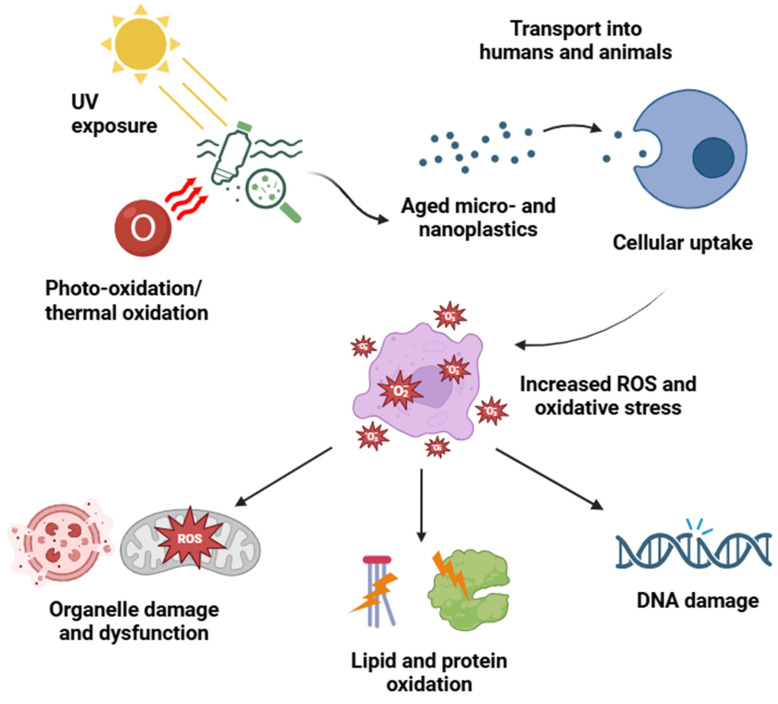
Micro- and nanoplastic (MNP) weathering process, cellular uptake, and consequent oxidative stress in cells. Plastic particles undergo aging through UV radiation and photo- and thermal oxidation, leading to structural changes and chemical alterations on the surface of the particles. When transported into humans and animals, these microscopic particles are distributed across systems and ultimately taken up by various cell types. MNP internalization is accompanied by the generation of free radicals inside cells, meaning elevated reactive oxygen species (ROS) levels and ensuing oxidative stress. The presence of MNPs within cells and consequent ROS overload damage organelles and impair their functions. At molecular levels, lipid and protein oxidation debilitates their structure and function, while oxidative DNA damage may give rise to mutations.

MNPs can trigger both extracellular and intracellular ROS generation with extracellular ROS generation predominantly related to the degree of “aging”. Plastics in the environment age or weather in several ways, including photo- and thermal oxidation, as well as UV radiation [32]. Weathering processes of all types cause chemical alterations on plastic’s surface [33] (Figure 1). Particularly, photo-oxidation or UV radiation mediates cross-linking reactions by abstracting a hydrogen atom from a biomolecule, creating a reactive site that can then bond with another chain [25,34]. Once generated, the free radicals can react with atmospheric oxygen, leading to the production of primary polymer peroxy radicals and then secondary polymer alkyl radicals [12,35]. The extracellular free radicals may be responsible for the remarkable increase in ROS following the cellular uptake of weathered plastic particles. Some studies have found a pronounced difference between the ROS production induced by weathered microplastics and fresh ones; however, there are no certain explanations that can be given for the molecular mechanisms responsible for this difference. A study by Pannetier et al. observed an increase in ROS production in fish treated with weathered MPs compared to virgin MPs, attributing the phenomenon to contaminants present within the samples that were internalized into the cell along with the microplastics [36]. However, other studies have concluded that the formation of free radicals due to photo-oxidation is the cause for the same [12]. Other weathering-related variables, such as the sharpness induced by photo-degradation and the subsequent ease of internalization into the cells, could also be viable culprits. However, this fails to provide a definitive explanation for the underlying molecular mechanisms and does not address contradictory observations [32]. Beyond autonomous ROS generation, MNPs interfere with mitochondria, the major producer of intracellular ROS, to induce physical damage to the energy-producing organelle and trigger excessive ROS generation [12].

The utilization of reactive oxygen species assays has advanced the understanding of ROS formation within cells after MNP exposure and internalization. These assays have been conducted in multiple cell lines and have facilitated a thorough evaluation of how MNP size ranges, exposure durations and concentrations influence oxidative interactions between the particles and cells. Notably, various cell varieties tested in vitro, including immune, corneal, conjunctival, kidney, lung, colon, leukemia, and testicular cell lines, consistently exhibit ROS formation in response to MNP exposure, with smaller particle sizes and higher concentrations generally correlating with heightened ROS production [12]. Beyond conventional factors such as concentration, size, and exposure duration, structural modifications in microplastics have emerged as critical determinants in influencing ROS formation during cellular interactions. For instance, research by Qingying et al. shows that the oxidative potential of polystyrene nanoplastics, ranging from 100 to 1000 nm, increases when these particles are internalized in A549 cell lines after being exposed to UV light for about 2 months [37]. In contrast, extended aging (around 1 year) of 100 µm PS-MPs has been observed to decrease ROS formation [32]. Increased activation of oxidative pathways by MPs likely stems from structural alterations induced by the UV light “aging” process, potentially leading to the sharpening of MP edges and an increase in surface roughness, both traits commonly associated with membrane damage and elevated ROS levels within internalized cell lines [38,39].

### 2.2. Cellular Strategies for Relieving Oxidative Stress

Oxidative stress refers to the disruption in the balance between intracellular ROS production and neutralization by the cell’s antioxidant systems [12]. These antioxidant systems comprise antioxidant compounds such as vitamins C, E, and D3 and various enzymatic pathways implicated in antioxidant production oxidant elimination [25,40]. For instance, superoxide dismutase (SOD) and catalase (CAT) enzymes are part of the ROS-neutralizing systems in innate immune cells that neutralize superoxide radical (O_2_^−^) and hydrogen peroxide (H_2_O_2_), respectively [25,41]. Due to their scavenger roles, SOD and CAT are often used as biomarkers for oxidative stress [25]. In addition to ROS overproduction, MNP exposure can also increase the levels of these enzymes, activating the antioxidation mechanisms to respond to excessive intracellular ROS. Oxidative stress is also associated with several ROS-mediated damage manifestations that include lipid peroxidation, protein oxidative modifications causing structural and functional changes, and DNA damage resulting in mutations [12,42] (Figure 1). Over time, this can lead to cellular dysfunction, senescence, and apoptosis, increasing the risk for diseases, e.g., neurodegenerative disorders, cardiovascular diseases, and aging [43,44,45,46].

Oxidative stress assays consistently demonstrate similar trends in MNP-exposed kidney, intestine, and gastric cell lines as well as lymphocytes [47,48,49,50,51]. For instance, Shi et al. conducted a study using CCK-8 assay to evaluate human lung epithelial A549 cells exposed to polystyrene nanoplastics and phthalate esters (prominent plasticizers). Their research highlighted a direct correlation between nanoplastic concentrations and oxidative stress. While low concentrations showed no significant impact on oxidative stress, higher concentrations evoked increased oxidative stress and reduced cell viability [37]. Studies have also shown that chronic exposure over 12 days induces greater oxidative stress compared to short-term exposure over 48 h [52]. Overall, oxidative stress assays commonly reveal mirrored changes in oxidative stress and ROS levels following internalization by various cell types. However, its severity is influenced by the size and duration of exposure to MNPs.

In intestinal Caco-2 cells, PS-NP exposure led to an irreversible internalization of the nanoplastics by the lysosomes, as well as alterations in the structures of vacuoles, mitochondria, and lysosomes within 24 h [53]. This organelle transformation was associated with increased transcriptional expression of superoxide dismutase 2 (SOD2) and heme oxygenase 1 (HO1), two protective genes against oxidative stress. In the stomach, MP-induced oxidative stress disrupts cellular metabolism, potentially impairing gastric function and integrity [54]. In kidney cells, exposure to MPs like polystyrene microplastics (PS-MPs) led to elevated ROS levels and decreased expression of SOD2 and catalase (CAT) [55], implying compromised cellular defense against oxidative damage. Variations in the expression levels of SOD and CAT largely depend on the duration of exposure to microplastics (MPs). Chronic exposure (8 weeks) resulted in increased expression of these antioxidant enzymes, whereas short-term exposure (72 h) led to a lower expression. This difference is likely due to an elevated stress response in cells subjected to prolonged exposure [54,55]. Similarly, MP-exposed liver and lung cells undergoing oxidative stress exhibit significant alterations in metabolic and proliferative activities [55]. In placental cells, the oxidative stress induced by MPs is particularly concerning due to potential implications for fetal development. Increased ROS levels in placental cells can lead to DNA damage, inflammation, and apoptosis, potentially affecting pregnancy outcomes [56].

The intrinsic potential of MPs to produce ROS shows inflammogenic potential in vivo or cytogenic potential in vitro [32]. Microplastics also show SOD (superoxide dismutase)-mimetic activity, turning superoxide radicals into hydrogen peroxide; however, further studies are required to establish this phenomenon [57]. Human bone marrow mesenchymal stem cells (BMMSCs) and adipose mesenchymal stem cells (AMSCs) have shown increased expression of GPX3 (which encodes the ROS scavenging system), suggesting that MNPs activate ROS scavenging response [58]. Interestingly, polystyrene nanoplastics or their degraded forms downregulated intracellular ROS levels in human BMMSCs [59]. Antioxidant response after MNP exposure has been documented in model organisms as well [25].

Reactive nitrogen species (RNS) are radicals derived from chemical reactions between any free radicals and nitric oxide (NO). The formation of RNS within MNP-treated cells is significantly less documented than ROS formation. However, studies have determined their induction to be a result of the high reactivity of nitrogen within the organic structure of certain plastics (polyamide) [60]. More specifically, the photoaging of nitrogen-containing microplastics is thought to initiate the formation of NO-containing radicals, which are theoretically capable of promoting RNS production and nitrosative stress in the cells [60]. Unfortunately, the MNP-induced formation of RNS remains a subject that is mostly speculative.

### 2.3. DNA Damage

The genotoxic effects of micro- and nanoplastics on cells involve oxidative DNA damage, which may be responsible for cell cycle arrest and may promote carcinogenicity [44,61]. Oxidatively damaged DNA most frequently harbors 8-oxo-7,8-dihydroguanine (8-oxoG), a modified base that mispairs adenine and thus has the capacity to induce mutations [62,63]. Limited studies on MNP effects on DNA damage did not show MNPs to directly interact with DNA as nuclear localization of the particles is not seen in the studies so far. There are studies demonstrating micronucleus formation in the presence of polyethylene microplastics (PE-MPs), possibly due to PE-MPs’ potential to be clastogens or having aneugenic potential but not yet established [48,64,65]. However, whether MNPs universally induce DNA damage is contested, as a handful of reports, mostly involving aquatic organism models, are available. Both in mussels and in the hemocytes of *S. plana*, breakage of DNA strands was observed following treatment with polystyrene and polyethylene microparticles (20 μm), respectively [66,67]. According to both studies, the observed DNA damage is interlinked with oxidative stress. Insights from broader nanotoxicology research indicate that nanoparticles can cause DNA damage via two mechanisms: direct and indirect [68]. Direct interaction of nanoparticles and DNA can be a cause of DNA damage while the production of ROS by nanoparticles can be an indirect cause. A study demonstrated the lengthening of the G0/G1 cell-cycle phase in NIH 3T3 cells following PS-NP treatment, potentially indicating checkpoint control activation following DNA damage [69]. Another study showed that PS-NPs only showed genotoxicity when used at higher doses but not otherwise [70]. Overall, DNA strand breaks can be induced by both MPs and NPs in a surface charge-dependent and size-dependent manner. Although the exact cause of DNA damage is yet to be fully understood, oxidative stress and physical interactions are highly likely to contribute to it. The levels of DNA damage markers, such as histone H2AX phosphorylation (γ-H2AX) showed elevation whereas p53-binding protein 1 (53BP1) showed lower expression post-exposure to PS-MPs [46]. Moreover, chromosomal abnormalities like breakage and dicentric chromosomes are also found in some cases [71]. Epigenetic changes and gene expression modulations associated with MNP exposure have started to be uncovered (for a review, see [72]). This could result in accelerated cell aging, inflammation, or induction of malignant transformation.

## 3. Cellular Impacts of Micro- and Nanoplastic Exposure

### 3.1. Effects on Cell Viability

Metabolism-based assays, such as MTT, MTS, or XTT assays, are often utilized as a direct evaluator of xenobiotic cytotoxicity in vitro. When applicable, such assays can measure the impacts of MP exposure on cell viability in cultured cells. Studies have demonstrated that human lung cell lines treated with polystyrene microplastics exhibit size- and concentration-dependent cytotoxicity. For instance, polystyrene nanoplastics (80 nm) demonstrate markedly higher cytotoxicity compared to larger particles (2 µm), with increasing concentrations leading to a progressive decline in cell viability [39,73]. Similar trends have been observed in various other cell types in vitro, including intestinal cells, lymphocytes, testicular cells, and osteoblasts. These studies consistently show that exposure to microplastics, regardless of the cell type, results in reduced cell viability and increased cytotoxicity [46,49,74,75]. There was a noticeable increase in the expression of pro-apoptotic genes, such as BCL2-associated agonist of cell death (Bad), and a corresponding decrease in BCL-2 expression in HK-2 human kidney cells, accounting for reduced cell viability [47]. These findings highlight the pervasive nature of microplastic-induced cytotoxicity and the importance of further research to understand the mechanisms underlying these effects.

Evidence of mitochondrial membrane potential depolarization and autophagosome formation implies that apoptosis and autophagy are simultaneously induced by polystyrene microplastics [76]. At low concentrations (3 ng/mL), inflammatory markers (a pool of 33 cytokines) are activated, whereas, at high concentrations (300 ng/mL), autophagy is induced [76]. In the case of immune cells, T cells appear to be least affected by microplastics, whereas phagocytic dendritic cells and macrophages show high sensitivity. While markers like CCL2, IL-17A, and IL-10 mostly increased, IL-6 levels were more dependent on the type and concentration of microplastics [77]. There is limited research on human cells on the impact of MNPs on senescence markers, but there is evidence of effects on senescence markers on rat lung tissue treated with microplastics. An increase in p16 and p21 was evident after eight weeks of treatment with microplastics (0.3 and 0.6 mg per week). Sphingosine levels decrease when treated with microplastics, demonstrating that microplastics can significantly hamper sphingolipid metabolism which in turn alters cellular apoptosis as macrophage phagocytosis is affected [78]. It has been shown that sharper-edged microplastics lead to lower cell viability in peripheral blood mononuclear cells, red blood cells, mast cells, human dermal fibroblasts (HDFs), and cervical cancer cells [38].

Microplastics are mostly uncharged in the environment, though it depends on the category of plastics and coating material used for desired products. However, in water when the microplastics are further degraded, they can have substantial charges [79,80]. When it comes to surface charges, cytotoxicity, Protein kinase A inhibition as well as cell cycle arrest were more aggravated by NH_2_-labeled PS-NPs than COOH-labeled ones [56]. Moreover, there was evidence of potentially irreversible damage to the acidic vacuolar compartment of intestinal cells (HT-29) [49]. In human lung cells, expression of endoplasmic reticulum (ER) stress proteins indicates that NH_2_-PS-MPs elevated ER stress through the PERK-EIF2α and ATF4-CHOP pathways [81]. Though polyethylene microplastics had minimal impact on cell viability in lung and intestinal cells, at the highest concentration of 1000 µg/mL, it triggered oxidative stress and reduced cell viability [82]. In human umbilical vein endothelial cells, short-term exposure (6 h) to 0.5 μm PS-MPs leads to a decline in tube formation through repression of vascular endothelial growth factor signaling [83]. Polystyrene microplastics reduced cellular proliferation and caused morphological changes in kidney and liver cells [55]. Microplastics also affected gene expression related to ECM- and integrin-mediated adhesion in human dermal fibroblast-derived spheroids.

### 3.2. Cell Type-Specific Effects

This section serves to discuss the cell type-specific effects of MNPs, grouping different cellular and organoid models under their shared tissue/organ of origin. In a lung carcinoma cell line (A549), MNPs, particularly polyvinyl chloride (PVC), have been shown to induce cellular senescence, demonstrated by elevated SA-β-gal positive cells, p16 and p21 proteins, and enhanced secretion of senescence-associated secretory phenotype (SASP) factors. This senescence is linked to ROS signaling, with antioxidant treatments significantly reversing these effects [84]. The detrimental impact of MPs extends to the structure and function of lung tissues, impairing physical function and inducing senescent cell accumulation. This is coupled with increased inflammatory cells and factors, chronic inflammation, and the potential development of premature emphysema [85].

In a liver organoid model, PS-MPs showed an elevated level of aspartate aminotransferase (AST) and alanine aminotransferase (ALT) after 48 h of treatment with an increase in IL-6 as well. PS-MP also promoted lipid accumulation in the liver organoids and induced hepatotoxicity by increasing HNF4A and CYP2E1 [86]. We previously showed that PS-MP lowered glycolytic activity in HepG2 liver cells [55].

Exposure of normal human embryonic kidney cells (HEK-293) to microplastic polymers such as cellophane, polystyrene, and polyamide exhibited increased apoptosis and cell detachment leading to cell death [87]. We showed that exposure to 1 µm polystyrene microplastics significantly reduced cellular proliferation in HEK293 cells without notable decreases in cell viability, which remained above 94% even at 100 µg/mL concentrations. However, morphological changes and internalization of microplastic particles were observed after 72 h, along with gene expression of glycolytic and antioxidant enzymes [55]. The uptake of microplastics correlated with concentration for polystyrene and size for polymethyl methacrylate (PMMA), with HEK293 cells showing increased uptake regardless of microplastic parameters [88]. Chen et al. confirmed the nephrotoxic potential of polystyrene microplastics using HEK293 cells. These microplastics adhered to and were engulfed by the cells, inducing cytotoxicity, triggering apoptosis and autophagy, reducing mitochondrial membrane potential, and forming autophagosomes. Non-cytotoxic concentrations activated inflammatory factors, while cytotoxic concentrations induced autophagy, reducing NLRP3 expression. PS-MP exposure of the cells also caused reduced expression of proteins that are implicated in maintaining barrier integrity in the kidney, promoting the risk of acute injury [76].

Vascular cells exposed to MPs exhibit cellular aging-related changes, including upregulated inflammatory factors and decreased lamin A, a key factor in vascular cell senescence. This degradation can be attributed to ROS-dependent activation of CDK5, highlighting a potential molecular mechanism for MP-induced cellular aging [89].

In skin cell models, nanoplastics are internalized in a time- and dose-dependent manner, causing the recruitment of gasdermin D (GSDMD) to stressed mitochondria and the activation of the AIM2 inflammasome, resulting in inflammation and senescence [90]. MPs promote cellular senescence in osteoblasts, as indicated by increased SA-β-gal positive cells and upregulated expression of p16INK4a and p21 [75].

**Figure 2:**
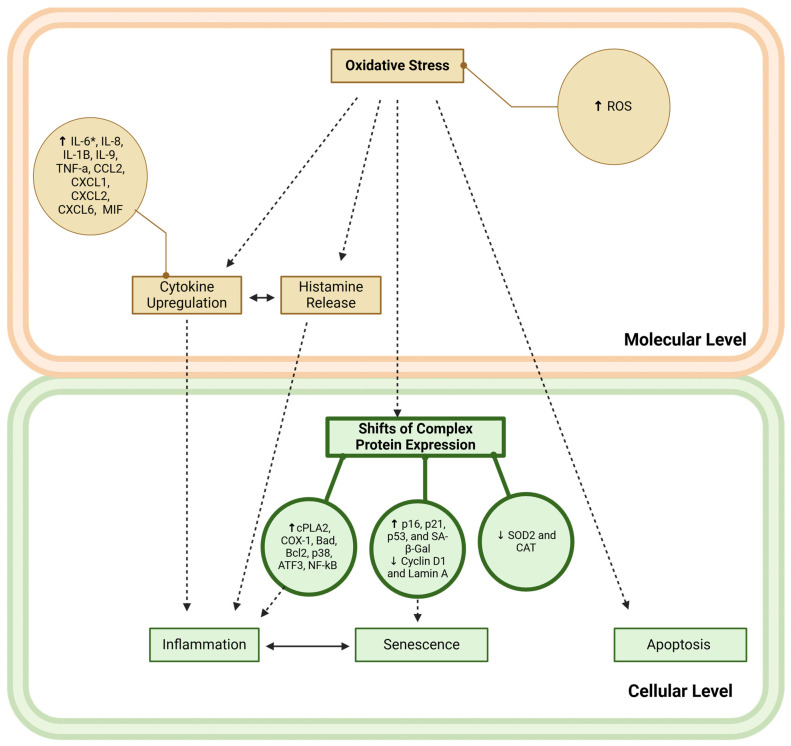
Molecular and cellular events leading to inflammation and senescence upon MNP exposure. Orange cube: key molecular consequences; orange sphere: detailed expression of relevant biomarkers; green cube: key cellular consequences; green sphere: detailed expression of relevant biomarkers; solid lines indicate the interconnectedness of biomarkers and key events; double-sided arrow: evidence suggests that key events are linked; dashed lines suggest that the key event may, in part, be a consequence of another key event; asterisk (*): indicates that IL-6 expression is downregulated in certain contexts due to microplastic exposure.

The next sections of the review discuss the implication of micro- and nanoplastics exposure in inflammatory responses, development, and senescence (Figure 2), potentially being the risk factors for aging and related diseases.

## 4. Activation of Inflammatory Pathways

Inflammation is a defense mechanism for the body against any pathogenic or foreign agents. The inflammatory process is triggered by toxicants or chemical compounds that are harmful to the body (xenobiotics) [91]. Micro- or nanoparticles can be categorized as “particulate xenobiotics” as they are found in living organisms and can interact with immune systems. The primary difference between xenobiotics and MNPs lies in their nature, with xenobiotics being chemical and MNPs being physical pollutants. MNPs being produced and used ubiquitously makes them more dangerous than xenobiotics. Nevertheless, MNPs can adsorb xenobiotics such as antibiotics [92]. The inflammatory process is a complex, multilayer mechanism that can often be destructive if overactivated [93]. Molecular, cellular, and tissue levels of the inflammatory process have been the focus of this article to present a clear view of the complex inflammatory mechanisms in the presence of MNPs.

Whether assessing pro-inflammatory or anti-inflammatory effects, enzyme-linked immunosorbent assays (ELISAs) and other immune response tests have assisted in piecing together the intricacies of molecular changes upon microplastic treatment in vitro. Exposure to microplastics consistently activates immune responses, notably inducing the expression of pro-inflammatory cytokines and histamine in human mast and microglial cell lines [38,94]. Among these cytokines, the proinflammatory cytokine interleukin (IL)-6 is frequently observed [95]. Studies have shown the expression of IL-6 in gastric, breast cancer, and liver cell lines, as well as keratinocytes [50,78,86,96,97,98,99], indicating a trend of pro-inflammatory responses induced by polystyrene microplastics. Other studies have documented the altered expression of other prominent inflammatory markers in response to MP exposure in vitro, including but not limited to ROS, nitric oxide (NO), tumor necrosis factor-alpha (TNFα), as well as IL-1β and IL-8 [7,50,76,78,94,96,97,98,99,100]. PS-NP exposure showed upregulation of IL6, IL8, and IL1β gene expression, with a negative impact on cell viability [101]. Similarly, Prietl et al. reported increased secretion of IL6 and IL8 when exposed to carboxyl PS-NPs in their study. This study demonstrated contrasting secretion patterns for IL6 and IL8, with IL6 levels increasing and IL8 levels decreasing, depending on the size of the microparticles used for treatment [102]. The possible reason could be that smaller-sized particles penetrate more easily, causing membrane damage and leading to increased cytokine production. However, decreased cell viability may result in lower cytokine secretion, and increased particle binding to proteins could also lead to reduced cytokine levels during measurement. Another study demonstrated increased mRNA levels of IL1α, IL1β, and IFN in exposed zebrafish [103]. Though the mechanisms involved in the induction of cytokine production in NP exposure are not yet understood, the observed responses could be interrelated to oxidative stress and lysosome membrane disintegration as discussed previously.

The other two levels of inflammatory responses induced by MNPs are cellular and tissue damage. Innate immune defense is comprised of many immune cells, which can interact with MNPs. In a fish model, degranulation of neutrophile granules and release of neutrophil extracellular trap (NET) is observed. This is evidence of NPs potentially stressing the innate immune system [104]. Neutrophil influx in the rat lung depends on the size of the micro- and nanoplastics (MNPs), with a greater influx observed when smaller particles (64 nm) are introduced. However, larger particles demonstrate increased secretion of interleukins, indicating the ability of MNPs to induce an inflammatory response [105].

Tissue damage is also evident across multiple cell models when treated with MNPs. Neutrophil infiltration extending to vacuolation and necrosis are found in the liver and gut of zebrafish when exposed to both PS-MPs (5 μm) and PS-NPs (70 nm). An increase in superoxide dismutase (SOD) and catalase (CAT) activity further suggests that oxidative stress may act as a primary causative factor in the initiation of the inflammatory response [106]. The evidence indicates that exposure to both microplastics (MPs) and nanoplastics (NPs) can induce inflammation, potentially mediated by oxidative stress and lysosomal dysfunction [107]. In mouse brains, oral ingestion of MPs showed that microglial cells (HMC-3 cells) engulfed MPs within 24 h of ingestion. There was a significant decrease in survivin levels (an anti-apoptotic marker) with a notable increase in pro-apoptotic proteins. Moreover, in PS-MP-treated human microglial cells, differentiation marker levels were altered with immune activation and apoptosis [94]. Yang et al. observed that NP-fed mice showed higher activation of IL1-producing gut macrophages than MP-fed ones, which affected the gut-brain axis and compromised cognitive function and memory in those mice [108].

Recent findings suggest that microplastics can stimulate the deregulation of cytokines linked to both pro-inflammatory responses and anti-inflammatory responses [109]. Notably, the extent and nature of inflammatory responses appear to depend not only on factors like the type and size of microplastics but also on their concentration. Different concentrations of the same microplastic can provoke varying inflammatory responses in identical test groups [76]. Weber et al. demonstrated that PVC (polyvinyl chloride) MPs triggered the secretion of both pro-inflammatory cytokines (IL-6, TNF) and anti-inflammatory cytokines (IL-10) in primary human monocytes possibly as a counter-balancing mechanism [110].

Table 1 lists the changes of inflammatory markers in MNP-exposed cells.

## 5. Micro- and Nanoplastics in Senescence: Implication for Development and Aging

Senescence generally refers to an irreversible cell-cycle arrest of damaged or unwanted cells, leading to a proliferation block and clearance by immune cells [118,119,120]. Acute senescence is a normal physiological process that accompanies organisms' development and wound healing to ensure the removal of unnecessary and abnormal cells [118]. Short-lived senescent cells are thus beneficial for organism growth, morphogenesis, and tissue repair. However, chronic or prolonged senescence causes dysfunction at the cellular and tissue levels and is an aging hallmark in adults [120,121]. As the role of MNPs in triggering senescence response in exposed cells continues to be explored, we discuss the key findings from the literature, considering both developmental and aging perspectives.

Since adult reproductive health dictates the development of offspring, how MNPs affect reproductive cells and organs in full-grown animals has also been a focus of research. A study by Liang et al. showed PS-NP-induced senescence of spermatogenic cells in mice as key reproductive damage, which mechanistically resulted from ROS enrichment in affected cells [122]. In a separate mice study, exposure to PS-MPs significantly increased cellular senescence markers such as β-galactosidase activity and p53-regulated cyclin-dependent kinase inhibitors (p21 and p16) in testicular cells, indicating an accelerated aging process. This was also accompanied by decreased cell proliferation and elevated levels of pro-inflammatory factors [46]. Another study revealed that maternal exposure to PS-NP during gestation and lactation disrupted NSC functions and affected neural cell composition and brain histology in the offspring [123]. For a further review of MNP reproductive toxicity and its impact on female fertility and progeny health, we suggest the following articles [124,125]. Multigenerational effects of MNPs have also been reported in aquatic and terrestrial animals [126,127].

Pluripotent and multipotent stem cells serve as surrogate models for studying the developmental impacts of MNP exposure. Self-renewal capacity and differentiation potential are of specific interest for these specialized cells. Past work by our group investigated the effects of PS-MPs on the neural development potential of human iPSC-derived cortical spheroids [128]. While we found that short-term MP exposure promoted proliferation and Nestin and PAX6 gene expression (two neural progenitor markers), long-term exposure affected the survival of the cells in the spheroid. The results imply that PS-MPs might interfere with human embryonic brain tissue development. A study on immortalized human neural stem cell lines demonstrated that PS-NPs caused cell death by apoptosis and decreased cell proliferation without being detected in the nucleus [129,130]. MPs of polyethylene terephthalate have been shown to alter the differentiation potential of human bone marrow mesenchymal stromal cells and adipose mesenchymal stromal cells when osteocyte and adipocyte derivation from the respective cells was attempted [131].

Aging is defined as the progressive decline in the function of organs and biological systems due to the gradual accumulation of damage and/or loss of damage response [121,132]. Damage and impaired damage response mechanisms are traditionally classified as aging hallmarks, which also explain the underlying processes and causative factors of aging [121,133]. Though senescence is a physiological damage response mechanism to eliminate dysfunctional cells, long-lived senescent cells can compromise tissue function and accelerate aging [118,120,134]. The functional decline also poses a significant risk for the development of aging-related diseases, including neurodegenerative and cardiovascular diseases [121,132].

**Figure 3:**
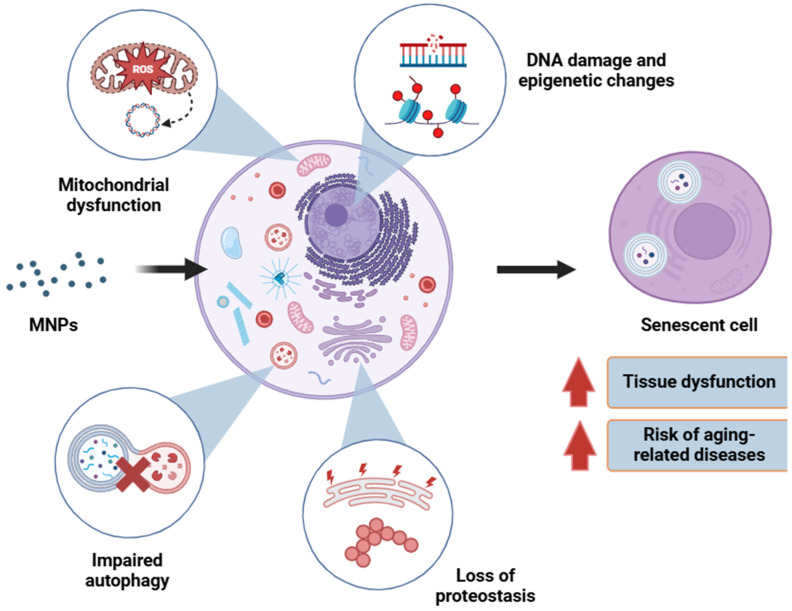
MNP exposure elicits the molecular hallmarks of aging in human and animal cells. Mitochondrial dysfunction (in the forms of excessive ROS generation, loss of membrane potential, and mitochondrial DNA leakage) and ROS-mediated damages such as impaired autophagy, loss of proteostasis, DNA damage, and chromatin modifications trigger senescence in cells. Long-term senescence is detrimental to normal tissue function and ensues aging-related disease manifestations.

Among the hallmarks of aging, senescence remains a central cellular hallmark [120,121]. In fact, senescence is promoted by some of the molecular hallmarks of aging, namely mitochondrial dysfunction, DNA damage accumulation, and epigenetic alteration [121,133]. Interestingly, all these molecular changes are frequently observed to result from MNP exposure [45,46,72,135]. Certain aging hallmarks, such as impaired autophagy and loss of proteostasis, are also directly related to the overwhelming oxidative stress that MNP exposure is capable of generating [136,137]. Altogether, it implies the existence of a mechanistic framework for MNP-induced senescence and aging phenomena (Figure 3). However, studies that systematically examined MNPs’ role as causative factors for aging-associated changes are limited. The use of primary cells from healthy and diseased old donors to investigate the aggravating role of the plastic particles is also lacking. Nevertheless, here we summarize the relevant literature that focused on aging features in MNP-exposed animals and cultured cells.

Wang et al. showed that NP-induced oxidative stress causes mitochondrial destabilization and leakage of mitochondrial DNA into cytoplasm in cardiomyocytes. This promotes inflammation and senescence responses via an activated cGAS-STING signaling pathway [45]. The changes are also accompanied by decreased heterochromatin marker H3K27me3 and increased DNA damage marker γ-H2AX [45]. In a mouse study, PS-MP treatment suppressed osteogenic ability in the animals by triggering osteoblast senescence in bone trabecula, likely through a mechanism that involves autophagy impairment in the senescent cells [75]. A separate mouse study demonstrated that environmentally relevant levels of MNPs can cause neurodegeneration, denoting a significant risk for even human brain health [138]. In endothelial cells, nanoplastic-induced senescence has been linked to an upregulation of sodium-glucose co-transporter, SGLT2, and inhibition of the co-transporter with a small-molecule inhibitor, enavogliflozin, significantly reduced senescence-associated markers [129]. Nanoplastics have been shown to cause oxidative damage to the zebrafish brain and elevate aging markers, culminating in learning and memory impairment [139]. Polyethylene MNP exposure in human vaginal keratinocytes led to altered DNA methyltransferase and ten-eleven translocation enzyme (TET) levels, implying subsequent epigenetic dysregulation and accelerated aging of the cells [135].

Table 2 lists the changes of senescence markers in MNP-exposed cells.

## 6. Perspectives and Future Directions

In recent years, the pervasive presence of micro- and nanoplastics (MNPs) in our environment has sparked concerns regarding their potential impacts on human and animal health [1]. MNPs’ persistent and overwhelming presence in the environment makes them more available for uptake by skin cells via direct contact, lung cells via respiration, and gastric cells via food and water intake. Mechanistically, MNPs are taken up by innate immune cells (e.g., macrophages) via phagocytosis and can be internalized via micro-pinocytosis and persorption across the intestinal epithelium. Our review aimed to summarize the effects of MNPs on oxidative stress, inflammation, and senescence, critical processes underlying various aging-related disease, revealing a complex interplay between MNP exposure and these biological processes.

Studies consistently demonstrate that exposure to MNPs leads to increased production of reactive oxygen species (ROS) across multiple experimental models, including cell lines, organoids, and animal systems. This elevation in ROS is indicative of oxidative stress, which can result in damage to cellular macromolecules such as DNA, proteins, and lipids, thereby contributing to cellular dysfunction and apoptosis [12,42].

The mechanism by which MNPs induce oxidative stress appears to be multifaceted. Physical characteristics of MNPs, such as size, shape, and surface area, as well as chemical additives and absorbed environmental pollutants, may enhance ROS generation. The presence of heavy metals and persistent organic pollutants adsorbed on MNP surfaces can exacerbate oxidative stress [140]. Previous studies indicate that smaller particles, due to their larger surface area-to-volume ratio, tend to produce more significant oxidative responses [141]. These insights are crucial for understanding how different types of MNPs might vary in their toxicological impacts. One caveat in the research arises from the overdependence on tumor cell lines to elucidate mechanisms of ROS production in vitro. While these cell lines offer ease of culture due to streamlined and well-characterized protocols, MNP stress response in healthy primary cells warrants further investigation. Overhauling ROS formation in vivo upon MNP exposure of live animals is also of considerable interest.

Beyond oxidative stress, there exists a clear link between MNP exposure and the upregulation of pro-inflammatory cytokines [97]. This inflammatory response is a common feature in the pathology of many diseases, including neurodegenerative disorders and cardiovascular diseases [25,26]. The increase in cytokines such as IL-6, TNF-α, and IL-1β suggests that MNPs may trigger immune responses, potentially through the activation of the NF-κB signaling pathway [46]. It is noteworthy that chronic inflammation is a hallmark of aging and age-related diseases. Thus, the pro-inflammatory effects of MNPs may not only contribute to immediate immune responses but also accelerate aging processes, a hypothesis that warrants further exploration.

MNP exposure is associated with the induction of cellular senescence, characterized by the increased expression of senescence-associated markers such as p21 and p16 [46,75,85,89,90]. Cellular senescence contributes to tissue dysfunction and the aging phenotype, and the observed senescence is likely a consequence of both oxidative stress and inflammation, creating a feedback loop that exacerbates cellular aging [142]. The aging process is closely linked to functional decline in various organ systems, so the acceleration of this process by environmental pollutants like MNPs could have far-reaching public health consequences. However, there remains a considerable knowledge gap regarding the effects of MNPs on old individuals, whether healthy or morbid. It can be overcome by including primary cells from aged donors in future aging-focused studies.

In conclusion, while the health impacts of MNPs are still being unraveled, the growing body of evidence suggests that MNPs are more than mere environmental pollutants. They actively modulate biological processes that could accelerate disease progression and aging. Addressing the challenges posed by MNPs will require a multidisciplinary approach, involving environmental scientists, toxicologists, healthcare professionals, and policymakers, to safeguard public health and the environment.

## Figures and Tables

**Table 1:** Effects of micro- and nanoplastics on inflammatory markers.

Particle (s)	Particle Sizes	Concentration and Duration of Exposure	Cell Lines	Relevant Inflammatory Biomarkers	References
PS	1.878 ± 0.677 μm	0.025, 0.05, 0.1, 0.2, 0.4 0.8 μg/mL, 120 min; 0.8 μg/mL, 0, 5, 10, 30, 60 min; 0.05, 0.1, 0.2, 0.4, and 0.8 mg/mL, 1 and 2 h	HK-2, kidney cells of 6 week old male C57BL/6 mice	Upregulation of cPLA2, COX-1, Bad, Bcl2, p38. ROS generation	[47]
HD-PE and LD-PE	1–10, 50, and 100 μm; 25–75 and 75–200 μm	10, 100 μg/mL and 1 mg/mL; 2 days for histamine release, 4 days for cytokine release	HMC-1	Upregulation of IL-6, TNF-α, and histamine	[38]
PS	3 and 10 µm	100–1600 particles/mL; 0.5, 1, 2, 3, 4, 5, 6, and 24 h; 7, 14, 21, 28, and 48 days	HT-29	Expression of ROS	[49]
PS	1–200 µm	1000, 100, 10 µg/mL	PBMC, Kato III, HeLa, HDFs	Upregulation of IL-6 and TNF-α	[39]
PP, PS	100 µm	200 mg/mL; 0.5, 1, 2, 3, 4, 5, 6, and 24 h	Caco-2, HepG2, THP-1	THP-1: Upregulation of IL-6, IL-8, IL-1β, TNF-α; ROS generation; Caco-2, HepG2: Upregulation of IL-6, IL-8, TNF-α	[32]
PS	1.72 ± 0.26 μm	1–1000 μg/cm^2^; 24 and 48 h	BEAS-2B	Upregulation of IL-6 and IL-8	[15]
PS, FRPS, PMMA	PS: 50, 200 nm and 1 μm. FRPS: 44 nm, 190 nm, and 1.04 μm PMMA: 70, 400 nm, and 1.1 μm	1, 10, and 100 μg/mL; 24, 72 h	A549, HEK293, HeLa	Upregulation of IL-6, IL-8, and TNF-α	[96]
PTFE	31.7 ± 5.6 μm and 6.0 ± 2.1 μm	10, 100, 500, 1000 μg/mL; 24 h, 48 h	A549, U937, THP-1, Jurkat, HaCaT	Upregulation of TNF-α and IL-6 in U937; downregulation of TNF-α in THP-1; upregulation of IL-6 in A549; mixed regulation of IL-6 in HaCaT depending on concentration	[99]
PS	213.7 ± 8.2 nm.	1, 10, 20, 50, 100 mg/L; 48 h	GES-1	Upregulation of IL-1β and IL-6	[50]
PS, F-PS HDPE, Nylon, Car tire, and Ocean Cleanup	PS: 0.05, 0.1, 1 and 10 μm. F-PS: 0.05, 1, 10. HDPE: 0–80. Nylon: 3 × 13 and 10 × 30 μm. Car tire: 0–120 μm Ocean cleanup: 0–40 μm.	1 mg/mL; 24, 48, 96 h	Human intestinal colon tissue and MucilAir™	Upregulation of IL-6 in MucilAir™	[111]
PS	4.8–5.8 μm	1 mg/mL; 6, 24, 48 h	HRT-18	Upregulation of IL-8	[97]
Fl-PS, PPS	20, 50, 100, 500 nm; 5, 10 μm.	1000 μg/mL; 24 h	Human umbilical vein endothelial cells	Upregulation of IL-1β, IL-6, and TNF-α	[78]
PA-12	1–5, 20–60 μm	100 μg/mL; 24 h, 14 d	PBMC-derived macrophages	Upregulation of IL-8	[98]
PP	16.4 μm	3–300 ng/mL for 24 h	MDA-MB-231 and MF-7	Upregulation of IL-6	[112]
PS	3.39 ± 0.30 μm	3–300 ng/mL; 24 h	HEK293	Upregulation of 33 inflammatory cytokines at 3 ng/mL, inhibition of NLRP-3 at 300 ng/mL, and reduction in inflammatory response	[76]
PS, NH_2_-labeled PS, PMMA	1 μm, 200, 50 nm	1, 100 μg/mL; 24, 72 h	PBMCs	Downregulation of IL-1β, IFN-γ; upregulation of CCL-2, IL-17A, IL-10; varying regulation of IL-6 depending on MP type and concentration	[77]
PS	1 μm	0.25, 2.5, and 25 μg/mL; 48 h	H1 ES, differentiated toward hepatic function	Upregulation of IL-6 and COL1A1	[86]
PS	0.2, 2, and 10 μm	1, 5, and 10 μg/mL; 24 h	HMC-3	Downregulation of IL-1β, CCL2, and TGF-β	[94]
Fluoresbrite- dyed MP particles	50 and 100 nm	0.008, 0.04, 0.2, 1, 5, and 10 mg/mL; 48 h.	Human Colon Fibroblasts (CCD18-Co)	Upregulation of IL1-Ra, CXCL1, MIF, serpin E1, and IL-8	[100]
PS	1 μm	5, 10, 25, 100 μg/mL; 48 h	Human umbilical vein endothelial cells	Upregulation of IL-6, IL-8, TNF, IL-1β, and MCP-1	[113]
NH_2_- and COOH-labeled PS	25, 50, 100, 500 nm	0, 20, 39, 78, 156, 313, 625, 1250, 2500, 5000 μg/mL; 24 h	JEG-3	Upregulation of CXCL6, ATF3, A20, and CCL by PS-NH_2_; upregulation of CXCL2, CXCL6, ATF3, and A20 by PS-COOH	[56]
PS	1 μm	0.25, 0.5, and 1 mg/mL; 24 h	TM4	Upregulation of NF-κB, IL-6, IL-8, and TNF-α	[46]
PE	30.5 ± 10.5 and 6.2 ± 2.0 μm	1–1000 μg/mL; HaCaT treated for 24 h, THP-1 and U937 treated for 48 h	HaCaT, THP-1, and U937	Upregulation of IL-6 in HaCaT; downregulation of IL-6 in Thp-1 and U937	[82]
PVC, ABS	25–200 µm	10, 100, 1000 µg/mL; 1, 5 d	HRBC	Upregulation of IL-5 and TNF-α	[109]
PS	1 μm	40 mg/kg; 24 h	HCAEC, HUVEC	Upregulation of TNF-α, IL-1β, and IL-6; downregulation of IL-8 and MCP-1	[89]
Fluorescence-labeled nanoplastics	N/A	0.1–1 mg/mL; 24 h	A549, BEAS-2B	Upregulation of IL-6, TNF-α, and IL-1β	[45]
PS	800 nm	10, 100, and 500 μg/mL; 96 h	A549	Upregulation of IL-8	[114]
PS	100 nm	10, 25, 50, 100 μg/mL; 24–72 h	HaCaT	Upregulation of AIM2, IL-6, IL-1β	[90]
PP, SFb, LFb	20 µm, 50 ± 26 µm, 200 ± 90 µm	10, 000 µg/L, 24 h	Gut of 18 week old *Danio rerio*	Upregulation of IL-1α, Expression of ROS	[115]
PE	10–150 µm	2, 20, 200 µg/L; 3 d/week for 5 weeks	Intestinal tissue of male, 5 week old SPF grade mice C57BL/6	Upregulation of IL-1α IL-2, IL-5, IL-6, IL-9, IP-10, and transcription factors TRL4, AP-1, and IRF5; upregulation of growth factor G-CSF	[116]
PE	1–10 µm	0.002, 0.2 µg/g/d; 31 days	Stool and colon tissue of 8 week old female ICR mice	Upregulation of IL-6, IL-8, IL-10, and IL-1β; downregulation of transcription factors NF-κB and ERK1	[117]

PS, Polystyrene; HD-PE, High-density Polyethylene; LD-PE, Low-density Polyethylene; PP, Polypropylene; FRPS, Fiber-reinforced polymers; PMMA, Polymethyl methacrylate; PTFE, Polytetrafluoroethylene; F-PS, Fluorinated polystyrene; PVC, Polyvinyl chloride; ABS, Acrylonitrile butadiene styrene; SFb, Short Polypropylene fibers; LFb, Long polypropylene fibers.

**Table 2:** Effects of micro- and nanoplastics on senescence markers.

Particle (s)	Particle Sizes	Particle Concentration and Length of Exposure	Cell Model or Tissue (s)	Senescence Markers	Species	References
PS	1 μm	0.25, 0.5, and 1 mg/mL; 24 h	TM4	Downregulation of Lamin A	Mice	[46]
PS	1 μm	0.3, 0.6, and 0.9 mg/mL	HCAEC, HUVEC	Upregulation of SA-β-gal, p16, p21	Human	[89]
Fluorophore-labeled nanoplastics	1–1000 nm	0.1–1 mg/mL; 0–90 min	H9c2, AC16	Upregulation of SA-β-gal by PE, PP, and PS; upregulation of p16 and p21 by PE and PVC	Rat, human	[45]
PS	800 nm	10, 100, and 500 μg/mL; 96 h	A549	Upregulation of Sa-β-gal, p21	Human	[114]
PS	100 nm	10, 50, and 100 μg/mL; 6, 12, and 24 h	HaCaT	Upregulation of Sa-β-gal, p16, p21, p53; downregulation of 53BP1 and Cyclin D1	Human	[90]
PE, PP, PS, PVC	PE: 6.5 µm–1 mm; PP: 6.5–100 µm; PS: 3–100 µm; PVC: 6–25 µm	0.5, 5 μg/mL; 24 h	A549, BEAS-2B	Upregulation of SA-β-gal, p16INK4a, and p21.	Human	[84]
PS	1 μm	1 and 5 mg/kg of ddH2O; minimum daily intake was 6 mL	8 week old C57 mice testis tissue	Upregulation of p21, p16, and p53	Mouse	[46]
PS, PS-NH_2_, PS-SO3H	5 μm	10 μg/mL; 48 h	MC3T3-E1	MC3T3-E1: Upregulation of p21 and p16	Mouse	[75]
PS	100 nm	0, 100, 200, and 400 μg/mL; 48 h	MLE12	Upregulation of Sa-β-gal, p16, p21, p53, and y-H2Ax Downregulation of 53BP1 and Cyclin D1	Mouse	[85]
NPs	1–1000 nm	3, 6, 10 mg/kg 2 d/week for 8 weeks	Mice heart tissue	Upregulation of p16, p21, p53	Mouse	[45]
PS	100 nm	10, 25, 50, 100 μg/mL; 24–72 h	JB6-C30	Upregulation of Sa-β-gal, p16, p21, p53; downregulation of 53BP1 and Cyclin D1	Mouse	[90]
PE, PP, PS, PVC	PE: 6.5 µm–1 mm; PP: 6.5–100 µm; PS: 3–100 µm; PVC: 6–25 µm	25 and 100 mg/kg of PVC/d for 8 days	BALB/c mice lung tissue	Upregulation of p21 by PVC	Mouse	[84]

PS, Polystyrene; PE, Polyethylene; PP, Polypropylene; PVC, Polyvinyl chloride.

## Data Availability

This is a review paper and no new datasets were generated. The datasets used and analyzed are published in this paper and cited from the references.
